# Frequency-Dependent Responses of Extensometers to Atmospheric Loading: Evidence from Geodynamical Observatory Jingyuan in the NE Margin of the Tibetan Plateau

**DOI:** 10.3390/s26144413

**Published:** 2026-07-11

**Authors:** Jinling Yang, Xiaolin Yang

**Affiliations:** 1 College of Geography and Oceanography, Minjiang University, Fuzhou 350108, China; yangjinling86@foxmail.com; 2 School of Architecture and Engineering, Jinggangshan University, Ji’an 343009, China

**Keywords:** extensometer, atmospheric pressure, frequency-dependent response, transfer function, Geodynamic Observatory Jingyuan, Tibetan Plateau

## Abstract

Extensometers housed in vaults are capable of resolving strain changes of less than a nanostrain; however, they are differently distorted by variations in barometric pressure at different frequencies, which may result in complicated strain noise. Therefore, how to quantify the barometric effects in different frequency bands is a perennial challenge in extensometric observation and research. Since the first strain observations at the Geodynamical Observatory Jingyuan (NW China) in 2007, the barometric effect has proven significant. Nevertheless, the frequency dependence and underlying mechanism of this effect remain unknown. With targeted research lacking at present, this study attempts to adopt the transfer function method to provide a systematic diagnosis. The results indicate that (1) the North–South (NS) component responds significantly to low-frequency pressure waves, with a slight phase shift leading the phenomenon; (2) the East–West (EW) component responds well to pressure waves in high-, medium-, and low-frequency bands, and the frequency dependence of the barometric coefficient spectrum and phase shift spectrum is strong; (3) the frequency-dependent response effect of the EW component may be related to factors such as horizontal pressure gradient force, topography, and fracture medium. These findings not only contribute to sub-frequency band correction of the barometric effect at the Geodynamical Observatory Jingyuan but also deepen our understanding of the ground–atmosphere coupling mechanism in the fault zone.

## 1. Introduction

Continuous measurements of strain by vault-housed extensometers have the advantage of having a high resolution (less than 10^−9^), wide frequency band (DC to tens of Hz), and good continuity. They can be used for the study of the Earth’s internal structure (e.g., normal modes, free core resonance) [1,2], Earth tides [3,4], tectonic movements [5], earthquake source physics [6], slow slip events [7], volcanic activities [8], and tsunami loading [9].

Although extensometers are always installed in relatively long tunnels (tens to thousands of meters) covered by rocky mountains, many factors such as sunshine [10], air temperature [11], barometric pressure [12,13,14,15,16,17,18,19,20,21,22], rainfall [23], and hydrology [24] can interfere with the measurement values. Among them, barometric pressure fluctuations (hereinafter referred to as pressure waves) are a ubiquitous and persistent source of non-tectonic noise. Moreover, under the combined impact of factors such as cave structure, geological structure, fracture/fault systems, topography, and crustal heterogeneity, the response of strain to pressure waves of different periods also exhibits frequency dependence [12,13,22,25]. Therefore, how to quantitatively clarify the barometric effects of strain measured by vault-housed extensometers in different frequency bands remains a major challenge in the field of continuous observation of crustal deformation.

In recent years, some geophysicists have adopted targeted methods to address this demanding but intriguing problem, including least squares method, linear regression analysis, wavelet transform, transfer function, and finite element modeling. For example, Mentes and Eperné-Pápai used linear regression to calculate the frequency dependence of the barometric response of a vault-housed extensometer at the Geodynamical Observatory Sopron in Hungary [25]. Their results showed that within a frequency band of 0.15–0.42 cycles per day (cpd), the barometric pressure coefficient increased exponentially with increasing frequency. Similarly, Di et al. used the wavelet transform to solve the barometric effect on the strain observed at the Geodynamical Observatory Changshu in East China at different frequencies [26]. As a result, they found that the barometric coefficients fluctuated significantly within the frequency band of 5.6–180 cpd. In contrast, the transfer function has higher frequency resolution than the linear regression and wavelet transform methods; thus, Otsuka used the transfer function method to analyze the low-frequency barometric effect on the strain measured at the Geodynamical Observatory Rokko-Tsurukabuto in Japan [12]. He found that the barometric coefficient spectra were relatively stable within the frequency band of 0.14–0.33 cpd. However, due to the influence of groundwater and fracture zone of the fault, the strain response significantly lags behind the pressure wave changes. Unfortunately, the above methods are unable to reveal the physical transfer mechanism between the barometric pressure and strain changes from the perspective of geodynamics. Thus, a few scholars have further resorted to numerical models, including boundary or finite elements, to quantitatively model the dynamic coupling characteristics between the strain and the pressure wave. For example, Yanagisawa used a 2D boundary element model to quantitatively characterize the influence of topography and cave shape on barometric effects at the Nokogiriyama Observatory in Japan [27]. Among others, Kroner et al. [14] and Gebauer et al. [19,20] systematically resolved the dynamics of the influence of topographic and lithologic features on barometric effects via refined 3D finite element models.

**Figure 1:**
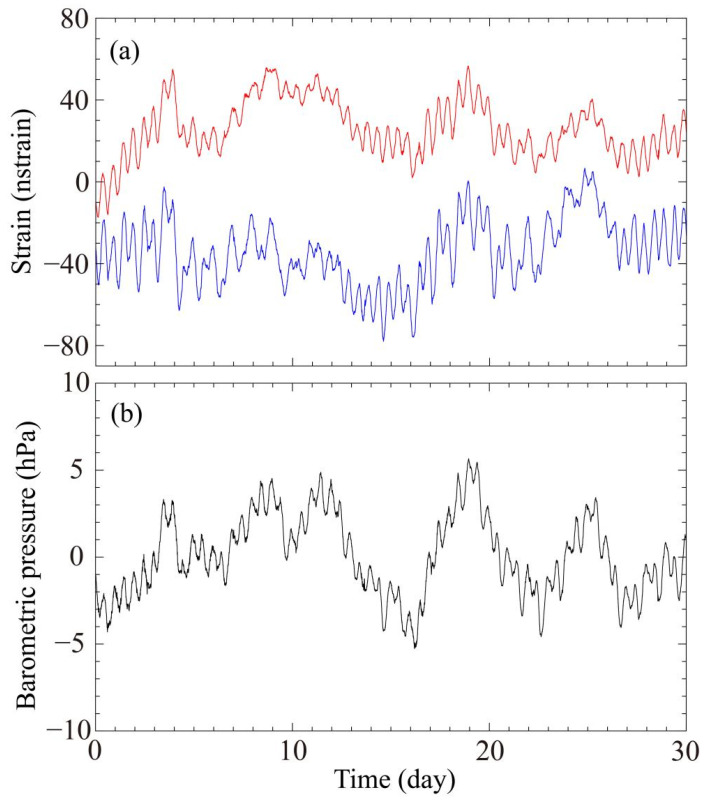
Variations in strain (extension negative) (**a**) and pressure wave (**b**) at the Geodynamical Observatory Jingyuan from 1 to 30 October 2023. The red and blue lines indicate the NS and EW components, respectively. Note: nstrain = 10^−9^.

The Geodynamical Observatory Jingyuan (GOJ) is located in the NE margin of the Tibetan Plateau (NETP). Due to the Indian–Eurasian collision since ~50 Myr, the NETP is one of the most tectonically active regions in the world [28]. Tectonically, this area is characterized by numerous active faults; therefore, the strain data observed at GOJ are very helpful for the study of earthquake precursors, regional tectonic movements, and crustal dynamics in NETP. However, the barometric effects are more significant in actual observations. Taking the extensometric records with one-minute sampling from 1 to 30 October 2023 as a showcase, the strain curves of the North–South (NS) and East–West (EW) components markedly and coherently fluctuated with low-frequency fluctuations in barometric pressure (Figure 1). It is not difficult to understand that the GOJ can effectively sense the pressure-induced crustal deformation. This gives rise to several questions, including: what are the characteristics of strain response at this observatory to pressure waves at different frequencies? What about its frequency dependence and response mechanism? Although these questions are undoubtedly worthy of deeper investigation in geodynamics, in-depth studies on GOJ are still scarce. In view of this, this study attempted to quantitatively analyze the frequency-dependent barometric response of GOJ using the transfer function. The relevant results not only contribute to the sub-frequency band correction of the barometric effect at the GOJ, but also advance the understanding of frequency-dependent responses of vault-housed extensometers to barometric pressure in fault zone.

## 2. Overview of the GOJ and Instruments

**Figure 2:**
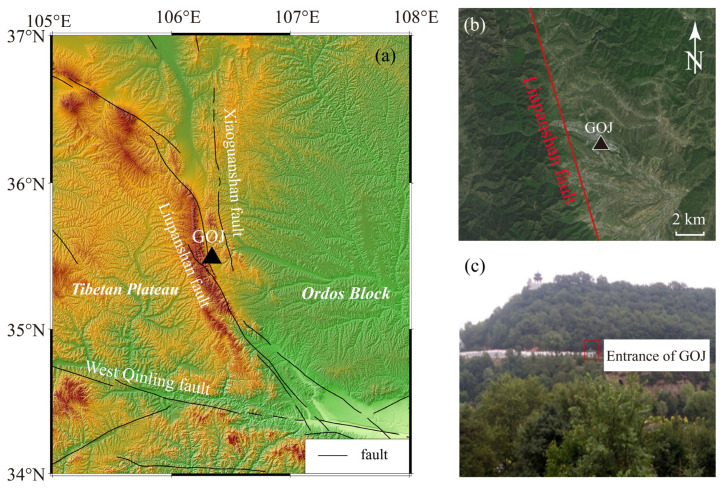
(**a**) Location of the GOJ (black triangle) and tectonic setting, (**b**) the surrounding mountains and fault, (**c**) a photo of the mountain coverage.

The GOJ (35.49° N, 106.34° E) is located in the air-raid shelter of Wolong Mountain in the southeast corner of Jingyuan County, Ningxia, with an elevation of about 1890 m. The lithology of the rock foundation is Quaternary pluvial sandy conglomerate. The rock mass is complete and dense, and the rock formation attitude is nearly horizontal. The overlay of the tunnel is about 15 m thick, and the trees on the hillside are relatively dense. The main structure of the tunnel is in the shape of an “L”, with a length of about 80 m. In the tectonic background, the GOJ is bounded by the Xiaoguanshan fault to the east and the Liupanshan fold-thrust belt to the west. The main lithologies of the strata in the area are sandstone, limestone, and mudstone [29]. The topography of GOJ and its surrounding areas is relatively complex. The western part is the Liupanshan Mountains, the eastern part is the loess hilly and gully area, and the central part is the valley and river channel area (Figure 2). Moreover, Jingyuan County belongs to a temperate semi-humid zone, and the monsoon climate and mountain vertical climate overlap in this area, with an average annual precipitation of about 642 mm and an average annual evaporation of about 1409 mm.

This SS-Y-type extensometer (manufactured by Wuhan Institute of Seismic Scientific Instruments Co., Ltd., Wuhan, China) was installed in March 2007. The length of invar-rod extensometers of the NS and EW components was approximately 22 m, with azimuths of 0° and 90°, respectively. Its resolution is less than 5 × 10^−10^ with a daily drift of <1 × 10^−8^. The type of the outdoor barometer was WYY-1 (manufactured by the National Institute of Natural Hazards, Ministry of Emergency Management of China, Beijing, China). The sampling interval for both instruments was one minute.

## 3. Data Selection and Pre-Processing

**Figure 3:**
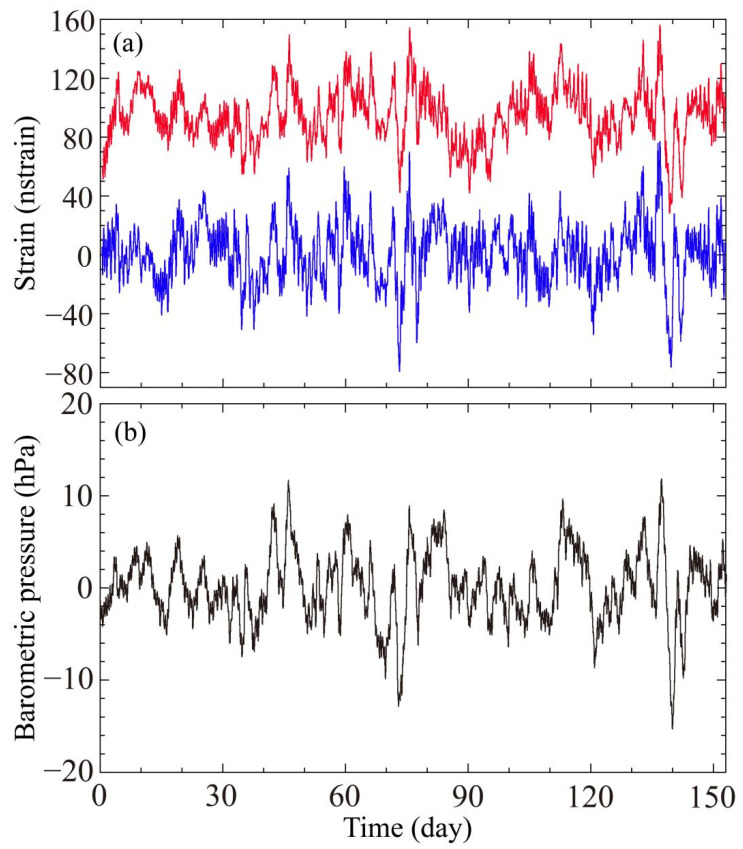
Time-varying curves of strains (**a**) and barometric pressures (**b**) from 1 October 2023 to 1 March 2024. The red and blue lines indicate the NS and EW components of strain, respectively.

To minimize the interference of rainfall, we mainly selected strain and outdoor barometric pressure measurements from 1 October 2023 to 1 March 2024, for a total of 153 days in autumn and winter. Linear interpolation was performed on the missing values in the observed data mainly due to the power outages. In addition, corrections were made for the step changes caused by calibration and observation system failures. Finally, we removed linear trends, annual variations, and seasonal variations from the time series curve. The final results after pre-processing are shown in Figure 3.

It can be clearly seen that the NS and EW components of strain exhibit similarities with the pressure wave; that is, strain varies approximately linearly with fluctuations in barometric pressure in the time domain. Their normalized correlation coefficients were as high as 0.7666 and 0.7665, respectively. This raises the following question: in the frequency domain, what are the characteristics and mechanisms of the response of strain measured at the GOJ to pressure waves at different periods? This will be the focus of the following investigation.

## 4. Methods

### 4.1. Coherence Function Method

It is worth noting that to ensure the physical significance of the transfer function, the correlation between barometric pressure and strain in the frequency domain first needs to be quantitatively assessed. If their correlation is low, the transfer function derived from this analysis loses its true physical significance. This value can be quantified using the coherence function $\gamma_{xy}^{2}$ [12,30]:

(1) $$\gamma_{xy}^{2}=\frac{{\left|G_{xy}(\omega )\right|}^{2}}{G_{xx}(\omega )G_{yy}(\omega )},\;(0\;\leq \;\gamma_{xy}^{2}\leq 1)$$

In Equation (1), *G_xx_*(*ω*) and *G_yy_*(*ω*) denote the auto-power spectra of the input signal *x* and the output signal *y*, respectively, while *G_xy_*(*ω*) denotes the cross-power spectrum. Physically, a coherence value $\gamma_{xy}^{2}$ = 1 indicates that the output signal *y* completely originates from the input signal *x*. In this case, the transfer system can be regarded as a linear system: if $\gamma_{xy}^{2}$ < 1, part of the output spectrum *G_yy_*(*ω*) at the frequency point comes from sources other than the input spectrum *G_xy_*(*ω*); finally, if $\gamma_{xy}^{2}$ = 0, it is implied that *x* and *y* are completely incoherent. From a statistical perspective, coherence values between 0.1 and 0.3, 0.3 and 0.5, and 0.5 and 1 indicate a weak, moderate, and strong correlation, respectively. Therefore, the transfer function has a given physical significance only when the coherence value is greater than 0.5. In practical calculations, the Hamming window was used as the window function in this study. The window length and step size were taken as 30 d and 15 d, respectively. Moreover, in the definition of the frequency band attributes, this study mainly refers to the classification criterion proposed by Lai et al. [31]. Specifically, *f* > 8 cpd, 0.5 cpd ≤ *f* ≤ 8 cpd, and *f* < 0.5 cpd denote high-, intermediate-, and low-frequency bands, respectively.

### 4.2. Transfer Function Method

Within the diurnal and semi-diurnal frequency bands, solid Earth tides (i.e., M_2_, N_2_, S_2_K_2_, K_1_, and O_1_) are significant input signals. To mitigate their interference on the transfer function, this study first implemented band-stop filtering without phase shift using second-order Butterworth filters on pressure waves and strain. This signal processing procedure can effectively remove solid Earth tide signals within frequency bands at 0.85–1.05 cpd and 1.85–2.05 cpd. After this filtering process, the transfer function *HB* of the response of strain measurement *y*(*t*) to the pressure wave *x*(*t*) at different frequencies can be expressed as

(2) $$HB(\omega )=\frac{BS(\omega )}{BB(\omega )}$$

(3) $$BS(\omega )=2\underset{t\to \infty }{\lim}\{\frac{1}{T}E[X^{*}(\omega ,T)Y(\omega ,T)]\}$$

(4) $$BB(\omega )=2\underset{t\to \infty }{\lim}\{\frac{1}{T}E[{\left|X(\omega ,T)\right|}^{2}]\}$$

where *BS*(*ω*) denotes the cross-power spectrum of the pressure wave and strain, and *BB*(*ω*) denotes the auto-power spectrum of the pressure wave. In addition, *T* denotes the finite time length, *E* denotes the mathematical expectation (i.e., the mean value), and *ω* denotes the angular frequency. *X*(*ω*) and *Y*(*ω*) are the Fourier transforms of the pressure wave and strain respectively, while X*(*ω*) is the conjugate of *X*(*ω*). The modulus |*HB*(*ω*)| and argument *Arg*[*HB*(*ω*)] of the transfer function are then the amplitude–frequency response (barometric coefficient spectrum) and phase–frequency response (phase shift spectrum) of strain to the pressure wave.

In practical digital signal processing and transfer function calculations, the whole observation data can be divided into multiple sub-datasets with a signal length of 2*^N^*, which has the advantage of improving the computational efficiency and obtaining more transfer functions. Then, the median value of each transfer function at the same frequency point is calculated one by one. With the above calculation process, the result error can be effectively reduced [31]. For the low-frequency band, we first used a second-order Butterworth filter to perform band-pass filtering without phase shift on the pressure wave and strain, respectively. This step filters out signals with a period of 1 h to 12 d. Then, sub-datasets with a duration of 2^16^ min (~45.5 d) were sequentially extracted and their power spectra and cross-power spectra were calculated. The window length and step size of the Hamming window were set to 2^14^ min (~11.4 d) and 2^13^ min (~5.7 d), respectively. For the intermediate-frequency band, we took 2^15^ min (~22.8 d) as a sub-dataset. The filtering range was 3 min~3 d, and the window length and step size of the Hamming window were set as 2^12^ min (~2.8 d) and 2^11^ min (~1.4 d), respectively. For the high-frequency band, the 2^14^ min data segment was used as a sub-dataset, and the filtering range was consistent with that of the mid-frequency band. The window length and step size of the Hamming window were set at 2^9^ min (~8.5 h) and 2^8^ min (~4.3 h), respectively.

## 5. Results and Mechanism Analysis

### 5.1. Coherence Function Results

**Figure 4:**
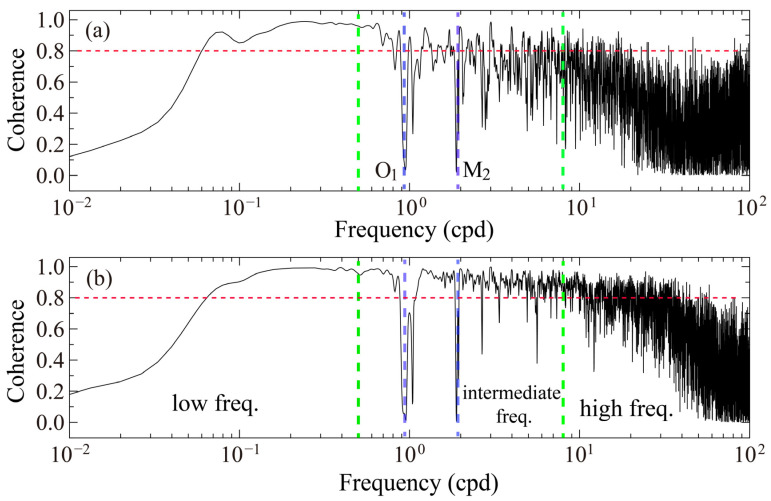
Coherence spectrum of NS (**a**) and EW (**b**) components for strain and pressure waves of different periods. The horizontal red dashed line means that the coherence value is equal to 0.8. The left and right vertical blue dashed lines indicate the O_1_ and M_2_ tidal constituents, respectively. The left and right vertical green dashed lines indicate 0.5 cpd and 8 cpd, respectively. Note that the threshold of coherence value is set as 0.8 here in order to obtain a more robust transfer function.

The coherence spectra of the NS and EW components of strain observed at the GOJ and the pressure wave are shown in Figure 4. To ensure the reliability of the transfer function, we set the threshold for the coherence value to 0.8. The following characteristics can be seen in Figure 4:

(1) Low-frequency band: The coherence spectra of the NS and EW components and pressure waves in the 0.01~0.5 cpd band showed overall frequency-dependent variation. In the 0.01~0.03 cpd band, the coherence increased slowly from roughly 0.20 to 0.30. By contrast, in the 0.03~0.1 cpd band, the coherence spectra all showed an accelerated increase, in which the peak coherence of the EW component reached 0.90. In the 0.1~0.5 cpd band, the coherence was greater than 0.80 as a whole, in which the NS and the EW components reached peak values of 0.98 and 0.99, respectively, at the 0.24 cpd and 0.39 cpd frequency points (Figure 4). This raises the question as to why the coherence spectrum of strain and pressure waves dropped rapidly in the frequency band less than 0.1 cpd? This may be due to the following two reasons: (a) The large fault systems, such as the West Qinling and Liupanshan faults, reduce the integrity of the Earth’s crust, which results in decoupling changes in the pressure wave loading field. (b) The data length of 153 days is relatively short (i.e., the seasonal signals have been removed after detrending). So why was the coherence spectrum in the 0.1~0.5 cpd band high? This was mainly due to the fact that the pressure wave loading in this frequency band is the main physical source of strain.
(2) Intermediate-frequency band: Within the diurnal and semi-diurnal frequency bands, the coherence of strain and the pressure wave showed a valley. The coherence of the EW component at the O_1_ frequency point was only 0.05 (Figure 4). An interesting question then is why the coherence within diurnal and semi-diurnal frequency bands suddenly drops. This is because in these bands, the tidal waves, such as M_2_, N_2_, S_2_, S_2_K_2_, S_1_, K_1_ and O_1_, have been removed from the strain and barometric pressure time series. This phenomenon also indirectly confirms the validity and reliability of the results of the coherence function in this work. A further comparison of the NS and EW components revealed that both exhibit significant fluctuation characteristics. The former had coherence values that were only 36% higher than 0.8, while the latter were 89% higher. These findings indicate that the NS component was weakly responsive to intermediate-frequency pressure waves, while the EW component was stronger.
(3) High-frequency band: The energy of pressure waves in this band was relatively small. In addition, factors such as instrument noise in strain and the barometric pressure observation system may also have had an impact. Therefore, the coherence values within this frequency band were usually low. As shown in Figure 4, most of the coherence values were significantly lower than 0.8. It is worth noting that in the 8~20 cpd band, the NS component only had coherence values higher than 0.8 at individual frequency points, while the EW component had 58% of coherence values greater than 0.8. The overall responses of strain at the GOJ to high-frequency pressure waves was weak, with the NS component being the weakest.

In general, the NS component of strain at the GOJ showed a better responsiveness to pressure waves in the 0.1~0.5 cpd band, while the EW component had a better responsiveness to the pressure waves in the 0.1~20 cpd band.

### 5.2. Transfer Function Results

**Figure 5:**
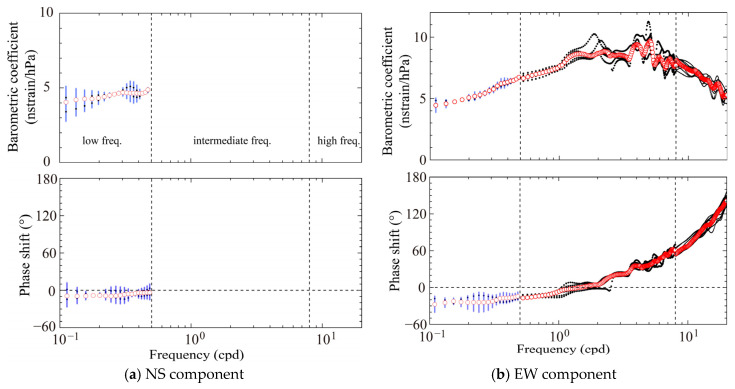
Transfer function (black dot) and its median value (red circle) for the response of strain to pressure waves at different frequencies. Note: a positive phase shift indicates that the strain lags behind the pressure wave, and vice versa. The left and right vertical black dashed lines indicate 0.5 cpd and 8 cpd, respectively. The horizontal black dashed line indicates zero-phase-shift. The blue vertical lines mark the 95% confidence intervals.

In view of the poor responsiveness of the NS component of strain at the GOJ to intermediate- and high-frequency pressure waves, only its transfer function in the low-frequency band was calculated in this study. Ultimately, we obtained a total of 11, 6, and 3 transfer functions in the high-, intermediate-, and low-frequency bands, respectively. The median values correspond to the barometric coefficient and phase shift, as shown in Figure 5. Next, this study will conduct a systematic diagnosis of its specific variation characteristics, while also elucidating its potential physical transfer mechanisms.

(1) NS component: In the low-frequency band, its barometric coefficient and phase shift were smoother overall, with variations ranging from 4.02 to 5.03 nstrain/hPa and −9.85° to −4.10°, respectively (Figure 5a). It is worth noting that both the barometric coefficient spectrum and the phase shift spectrum rose slowly with increasing frequency. The former may be due to the fact that the equivalent elastic strength of the crustal medium was increasing with the expansion of the neighboring area of GOJ, while the latter may be due to the variability of the horizontal pressure gradient force at different spatial scales [14,25].
(2) EW component: In the low-frequency band, the barometric coefficient spectrum and the phase shift spectrum roughly showed a linear increase with increasing frequency. Their variation ranges were between 4.44 and 6.72 nstrain/hPa and −27.03° and −14.05°, respectively (Figure 5b). This raises the question as to why the EW component exhibited a substantial leading response in the low-frequency band. This may be related to the complex topography and terrain gradient in the EW direction of GOJ (Figure 2) [19,20]. In the intermediate-frequency band, the barometric coefficient spectrum showed a roughly parabolic variation pattern with increasing frequency. Its variation range was between 6.63 and 9.67 nstrain/hPa, in which the peak of the barometric coefficient occurred at the frequency point of 5.05 cpd. The barometric coefficients at the frequency points of *f* = 4 cpd and 5 cpd both showed a sudden rise. This is most likely due to the S_4_ and S_5_ tidal waves in the atmosphere. For the phase shift spectrum, its spectral curve showed a roughly exponential gradual increase with increasing frequency. Variation ranged from −16.96° to 62.16°, in which the phase shift value turned from negative to positive at a frequency point around 1.45 cpd. With regard to why the EW component of strain lagged significantly behind the pressure wave in the intermediate-frequency band, this may be due to the complex mechanical characteristics of the fracture/fault zone around the GOJ [12]. In the high-frequency band, the barometric coefficient spectrum changed linearly downward with increasing frequency, while the phase shift spectrum changed exponentially upward. The variation intervals of the two were 5.09~8.11 nstrain/hPa and 54.81°~141.16°, respectively. So, why did the barometric coefficient spectrum fall rapidly while the phase shift spectrum rose rapidly? Figure 2 shows that the GOJ and its neighboring areas were mainly located in the fracture/fault zone. This indicates that the in situ domain and its surrounding media were more fragmented than the periphery. Therefore, at a small scale, the mechanical properties of its response to pressure waves were also more complex [12]. Overall, the EW component exhibited significant frequency dependence for its response to pressure waves at different periods. In the intermediate- and high-frequency bands, the barometric effect was more prominent.

In general, the NS component of strain at GOJ responded more significantly to pressure waves in the low-frequency band, while the EW component responded well to pressure waves in all the high-, intermediate-, and low-frequency bands. The frequency-dependent response effect was particularly prominent for the EW component.

## 6. Conclusions and Perspectives

To systematically elucidate the potential mechanism of the response of strain at the GOJ to pressure waves at different periods, this study conducted an in-depth analysis by adopting the coherence function and transfer function methods. The main results were as follows:

(1) Compared with the intermediate- and high-frequency bands, the NS component showed a better responsiveness to low-frequency pressure waves. Its barometric coefficients and phase shifts in the low-frequency bands changed in the ranges of 4.02~5.03 nstrain/hPa and −9.85°~−4.10°, respectively. The phase shift may have shown a minor leading response due to the horizontal pressure gradient force.
(2) In the frequency band of 0.1~20 cpd, the EW component showed a better responsiveness to the pressure wave. Its barometric coefficient spectrum and phase shift spectrum roughly showed “∧” and exponential variations with an increasing frequency, respectively. Their variation ranges were 4.44~9.67 nstrain/hPa and −27.03°~141.16°, respectively. This typical frequency-dependent variation indicates that the EW component’s response to the pressure waves exhibited strong frequency dependence, with a response mechanism that was also more complex.
(3) In the low-frequency band, the EW component exhibited a more pronounced leading response to pressure waves. In the intermediate- and high-frequency bands, the EW component lagged significantly behind the pressure wave. The former may be due to the complex topography in the EW direction of GOJ, while the latter may be caused by the relatively fragmented media in the observatory area and surrounding areas.

Although this study systematically reveals the frequency-dependent response features of strain at the GOJ on pressure waves, the mechanisms underlying this dynamic mechanism remain unclear at empirical and qualitative levels. As such, the stratigraphic structure and barometric pressure field at the GOJ and its surrounding areas warrant further investigation in subsequent dynamics studies. In addition, the dynamics of the regional barometric pressure field causing strains at the GOJ should be quantitatively simulated with the help of a 3D finite element model containing the real topography and the fracture/fault zones.

Moreover, plenty of external factors, such as rainfall, snow, temperature, and humidity, can change the mechanical property of the near-surface; accordingly, the atmospheric responses may vary with the change in season. Therefore, the relationship between strain and atmospheric loading in the frequency domain may considerably vary in other seasons (i.e., rainy season). More importantly, we should note that the present transfer functions, derived from an autumn–winter period, should not be directly assumed or used to represent other seasons without further validation. In a future study, we need to comprehensively examine the nature of atmospheric responses at the GOJ in different seasons.

Finally, it is also worth noting that the number of strain observatories in mainland China currently stands at 111 [32]. In this respect, several questions remain to be answered: What is the frequency dependence of the response of these observatories to pressure waves? What are the similarities and differences in the physical mechanisms underlying them? Undoubtedly, these are scientific questions worth exploring systematically, whose answers will help to improve the scientific robustness of site selection, instrument deployment, and barometric effect correction in the extensometer network of China.

## Data Availability

The extensometric and atmospheric pressure data used in this study can be downloaded at “zenodo” (https://zenodo.org/records/17614926, accessed on 8 July 2026).
